# Lipoid Proteinosis: A Rare Encounter in Dental Office

**DOI:** 10.1155/2015/670369

**Published:** 2015-03-19

**Authors:** Prasannasrinivas Deshpande, Mahima Veeranna Guledgud, Karthikeya Patil, Usha Hegde, Ankita Sahni, Sreeshlya Huchanahalli Sheshanna

**Affiliations:** ^1^Department of Oral Medicine & Radiology, JSS Dental College and Hospital, JSS University, Mysore, Karnataka 570015, India; ^2^Department of Oral Pathology and Microbiology, JSS Dental College and Hospital, JSS University, Mysore, Karnataka 570015, India

## Abstract

Lipoid proteinosis is a sporadic congenital metabolic disorder which is characterized by deposition of hyaline material in dermis, submucosal connective tissue, and various internal organs. It has an extremely low prevalence rate with less than 300 cases reported so far. This progressive disease has a vast spectrum of manifestations ranging from asymptomatic lesions to fatal seizures and respiratory obstruction making timely diagnosis of this rare disorder an imperative task for oral health care practitioners. We report a case of characteristic oral manifestations of lipoid proteinosis in a 28-year-old male patient along with a review of relevant prevailing literature.

## 1. Introduction

Lipoid proteinosis is a rare autosomal recessive congenital disorder having a vast continuum of ophthalmic, cutaneous, neurologic, and oral manifestations characterized by deposition of hyaline material in various organs. A review of the prevailing literature revealed that less than 300 cases have been reported so far and very few cases have been accounted from the Indian subcontinent [1]. Lack of profound knowledge and awareness makes diagnosis of this rare disorder an exigent task for oral health care practitioners.

Here we report a case of lipoid proteinosis in a 28-year-old male patient with accentuated oral manifestations along with its scrupulous diagnostic work up.

## 2. Case Discussion

A 28-year-old male patient, a single child from a consanguineous marriage, reported to us for a routine dental checkup. He presented with hoarseness of voice since childhood. He gave history of occasional recurrent self-limiting oral ulcerations since 2 years. He gave no history of seizure episodes, visual disturbances, or any other systemic symptoms. Patient's family and medical history were noncontributory.

**Figure 1:**
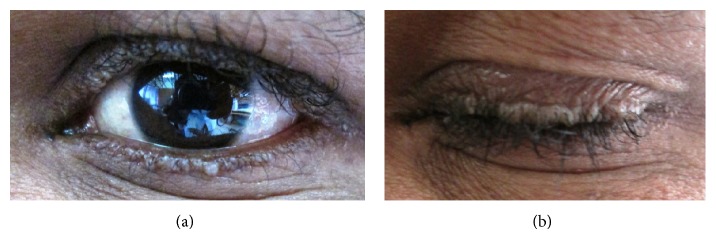
Waxy beaded papules on the margins of the eyelids.

On general physical examination he was found to be moderately built and nourished. There was presence of waxy beaded papules on the margins of both eyelids (Figure 1) and areas of hyperkeratosis on the dorsal surface of the hands.

**Figure 2:**
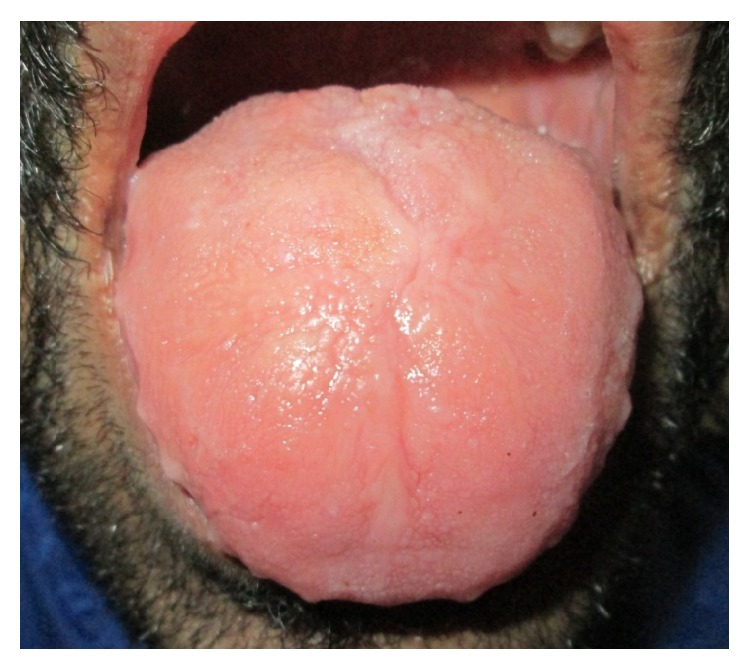
Macroglossia with crenations along the margins and loss of papillae on dorsum surface of the tongue.

**Figure 3:**
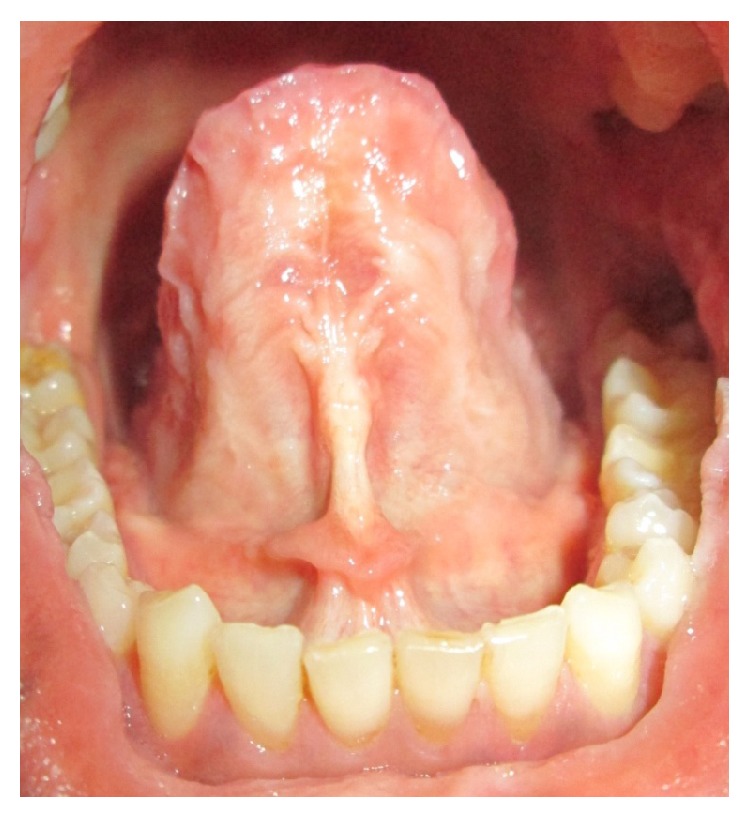
Sublingual frenum was stiff.

On intraoral examination, macroglossia was evident with crenations along the margins and there was absence of papillae on the dorsum surface. On palpation the tongue and the sublingual frenum were found to be stiff and limited mobility of the tongue was noted (Figures 2 and 3).

**Figure 4:**
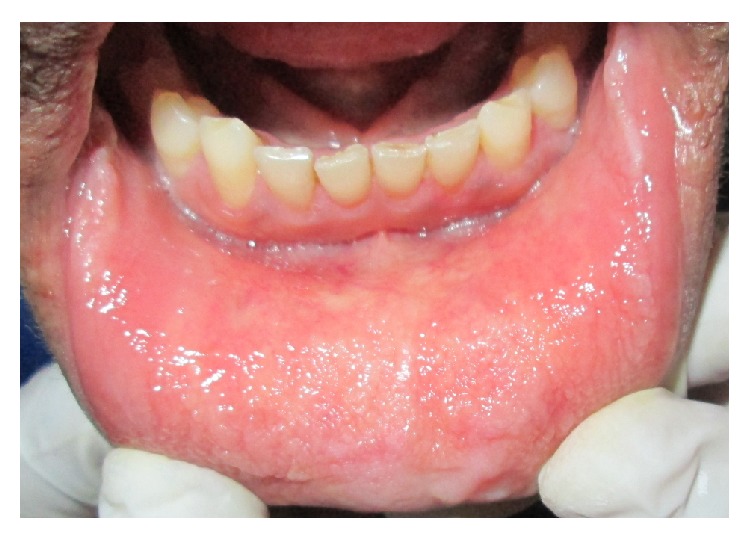
Diffuse yellowish waxy papular lesions were noted throughout the oral mucosa.

There was presence of diffuse yellowish waxy papular lesions throughout the oral mucous membrane which appeared nodular and rough on palpation (Figure 4).

A lateral skull view and posterioanterior view did not delineate the presence of any calcifications. On the basis of history and clinical appearance differential diagnoses considered were amyloidosis, lipoid proteinosis, myxoedema, and erythropoietic protoporphyria.

**Figure 5:**
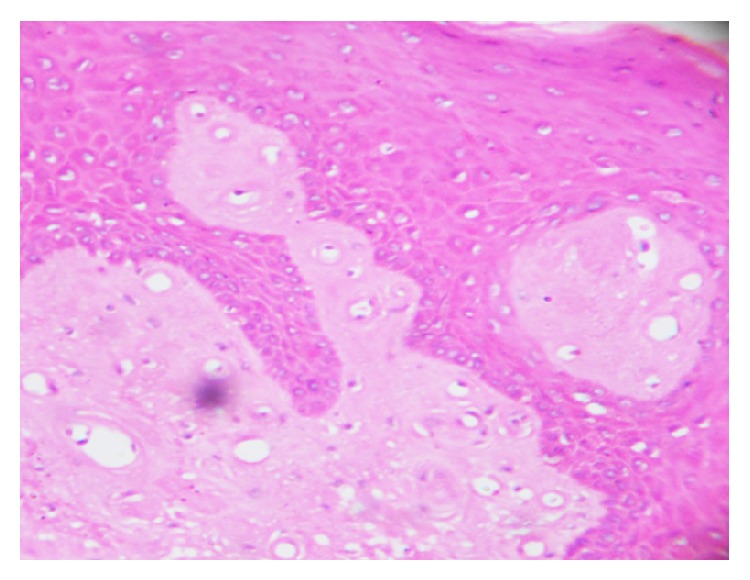
Hematoxylin and eosin stained sections (40x) revealed stratified squamous parakeratinized hyperplastic epithelium with presence of fibers, fibroblasts, blood vessels, and numerous eosinophilic amorphous aggregates in the connective tissue.

**Figure 6:**
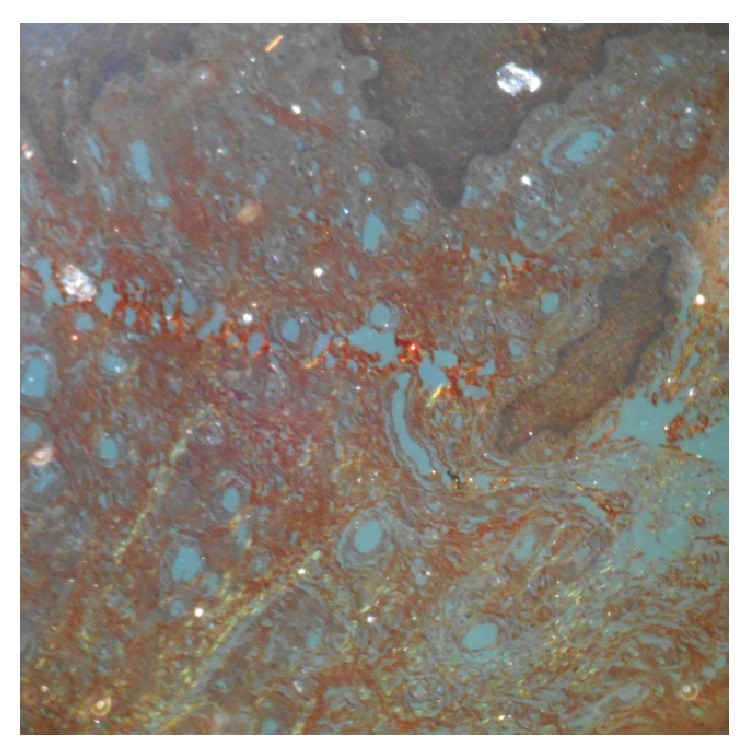
Congo red staining was negative.

**Figure 7:**
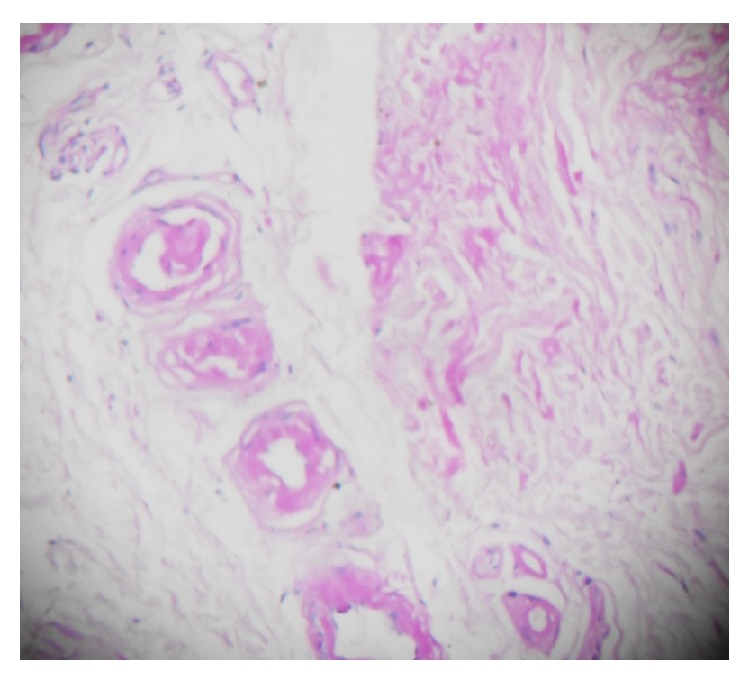
PAS staining showed positive results.

Complete hemogram and thyroid profile values were found to be within the normal range. An incisional biopsy from the buccal mucosa and ventral surface of the tongue was performed. Similar histological findings and staining patterns were observed in the sections from both sites. Hematoxylin and eosin stained sections revealed stratified squamous parakeratinized epithelium and it was hyperplastic in nature. The tongue specimen showed spongiosis of epithelial cells in few areas. The connective tissue showed hyalinized fibrous stroma with fibers, fibroblasts, blood vessels, and proliferating endothelial cells. Numerous eosinophilic amorphous aggregates were seen in the connective tissue (Figure 5). Similar type of deposit was also found around the blood vessels and the proliferating endothelial cells. Special staining with Congo red and crystal violet was found to be negative which ruled out the presence of amyloid tissue (Figure 6). Periodic acid Schiff staining (PAS) was found to be positive, which showed deposition of pink hyaline PAS-positive material in the connective tissue and around the blood vessels (Figure 7).

After clinicopathologic correlation a final diagnosis of lipoid proteinosis was rendered. Patient was educated and made sentient about his condition; however patient was lost to followup.

## 3. Discussion

Lipoid proteinosis also known as Urbach–Wiethe syndrome or Hyalinosis cutis et mucosae is a sporadic autosomal recessive metabolic disorder which was first described by Viennese E. Urbach and C. Wiethe in 1929 [2]. The autosomal recessive nature of this condition is exemplified by an increase in consanguineous marriages as was the case in the present patient [1].

Exact etiology of this disorder still remains an enigma; however some researchers believe that frame shift and nonsense mutation in the gene encoding for extracellular protein 1 (ECM 1) gene on band 1q21 are liable. ECM is a glycoprotein expressed in several tissues, including those of the skin. This glycoprotein is composed of two alternatively spliced isoforms, ECM1a and ECM1b, the latter lacking exon 7 of the 10-exon gene. Exons 6 and 7 are the most common sites for ECM1 mutations in LP [3]. Exact role of ECM1 gene is unclear, although some believe that it plays a pivotal role in skin physiology and homeostasis [4]. The corollary of this mutation is deposition of hyaline material in dermis and thickening of the skin and mucous and basement membrane around the blood vessels and adnexal epithelia [5]. Clinically, mutations outside exon 7 are usually associated with a slightly more severe mucocutaneous LP phenotype [3]. The pathology is also related to an alteration in the synthesis and metabolism of the collagen, leading to an increase in the production of collagen types IV and V by the endothelial cells of the blood vessels, a decrease in the production of collagen types I and II, and an increase in the synthesis of a glycoprotein substance by the fibroblasts [6, 7]. The decrease in the mRNA for type I procollagen and increase in mRNA for type IV procollagen result in underproduction of fibrous collagens and an overproduction of basement collagens, which tends to deposit in the skin and various organs. The disease is also postulated to be the result of lysosomal storage disorder involving multiple enzyme defects [7].

The earliest and most unswerving clinical sign of this disorder is hoarseness of voice due to hyaline deposition in laryngeal mucosa which may manifest at birth or later in life. Hoarseness of our patient's voice was the first manifestation which caught our attention. Oral health care practitioners who are not well aware of this feature may erroneously diagnose it as chronic laryngitis and overlook this vital clue [4].

Cutaneous lesions manifest as yellowish waxy areas commonly affecting face, lips, and margins of the eyelids [8]. Beaded papules over the palpebral margins of the eyelids identified as “Moniliform blepharosis” are pathognomonic for this disease [9]. Eye papules were characterized in the present patient and further strengthened our suspicion of lipoid proteinosis. Hyaline depositions in the conjunctiva, cornea, and retina may lead to corneal opacities or secondary glaucoma [10].

Areas like palms, axillae, knee, and elbows which are subjected to constant mechanical trauma may develop a hyperkeratotic, verrucous surface [11].

Intraoral manifestations include macroglossia, macrocheilia with fissuring, and nodular thickened mucosa due to infiltration of waxy yellowish white plaques and nodules. Loss of papillae of the tongue along with its restricted mobility has been reported in literature [11]. These clinical manifestations are consistent with the findings of the present case.

Intracranial calcifications have been reported with epilepsy, mental dysfunction, and neuropsychiatric abnormalities [12].

Clinical diagnosis is confirmed by histopathological examination. Histologic H and E stained sections divulge the presence of amorphous, extracellular, and eosinophilic hyaline material. They are PAS positive and diastase resistant [8].

In the present case complete haemogram and thyroid profile values were found within the normal range which ruled out the possibility of myxoedema. Congo red staining was found to be negative, which ruled out amyloidosis. Erythropoietic protoporphyria exhibits similar cutaneous clinical manifestations but absence of similar oral presentation ruled out the possibility of this disease. All in all, the clinical and histologic features of the present case favored a diagnosis of lipoid proteinosis.

No specific treatment is available for this uncommon disorder. Oral steroids, dimethylsuphoxide, intralesional heparin, etretinate, and penicillamine have been used in therapeutic management. Dermabrasion, chemical skin peeling, blepharoplasty, and Co_2_ laser therapy have also been suggested by a few authors [11, 12].

Even though it is a progressive disease, prognosis is good with a normal life expectancy [12]. However, patient must be habitually evaluated to evade any untoward life-threatening complication.

## 4. Conclusion

In a country like India where certain communities still have frequent consanguineous marriages, the probability of offsprings to be affected by this genetic disorder is high. Vast spectrum of manifestations of this sporadic genetic disorder may result in diagnostic ambiguity. Hence, it is imperative for oral health care practitioners to have profound knowledge to thwart erroneous diagnosis and render appropriate counseling and treatment to such patients.
